# The Occupational Therapy Profession in Saudi Arabia

**DOI:** 10.1155/2024/9982661

**Published:** 2024-01-17

**Authors:** Naif Qasem Aljabri, Kim Bulkeley, Anne Cusick

**Affiliations:** ^1^College of Medical Rehabilitation Sciences, Taibah University, Al-Madinah, Saudi Arabia; ^2^Faculty of Medicine and Health, The University of Sydney, Sydney, Australia

## Abstract

**Objective:**

To provide an evidence-based description of how the occupational therapy profession operates in Saudi Arabia.

**Methods:**

A case study methodology set out an evidence-based description of occupational therapy in Saudi Arabia. Three procedures were used: (1) a structured narrative review of publications on occupational therapy in Saudi Arabia based on searches of seven healthcare databases; (2) an audit of “grey literature” about or referring to occupational therapy education, practice, research, or the profession in Saudi Arabia using Google Scholar, Google search, official documents and websites, and relevant global organisation such as the WFOT website and documents; and (3) consultation with occupational therapists to further discuss emerging evidence from peer-reviewed articles and grey literature about occupational therapy in Saudi Arabia. Data were collected in Arabic and English. *Findings*. Occupational therapy in Saudi Arabia is a rapidly growing and emerging profession primarily focused on rehabilitation practice. Between 2010 and 2019, seven academic organisations commenced bachelor's degree programmes. Occupational therapy services in Saudi Arabia are concentrated in city-based secondary and tertiary healthcare services targeting rehabilitation and disability support. The practice aims to promote the reduction in impairment and increase activity performance and participation in valued life roles. Despite the increasing demand for occupational therapy services, there needs to be more access to and availability of their services. Enhancement of the awareness of occupational therapy among the public and other healthcare professionals is needed. A consistent definition of occupational therapy in Saudi Arabia would strengthen the profession, along with a national scope of practice, practice guidelines, increased workforce and development, and professional education information.

**Conclusion:**

Occupational therapy in Saudi Arabia is growing regarding the size of the workforce and the number of programmes and services provided, thus contributing to the health and well-being of the population primarily through rehabilitation. Further development of the profession is necessary.

## 1. Background

Occupational therapy (OT) is a patient/client-centred health profession focused on the promotion of health and well-being in meaningful human activities [1]. The primary aim of OT is to enable people to reduce impairment and enable them to engage in activities and roles they expect, want, or need to do by modifying either the environment or the activity itself [1]. Techniques are used to assess, treat, and follow-up patients to improve performance and satisfaction in routine activities of daily living (ADL), such as eating or dressing; instrumental ADL, such as shopping or moving about the community; and participation in meaningful and culturally important life situations, such as family gatherings in the community [2]. Across the world, occupational therapists (OTs) work with individuals across the lifespan, families, and communities in primary, secondary, and tertiary healthcare settings [2]. OT services have been offered in Saudi Arabia since the late 1980s and early 1990s, coinciding with the emergence of the modern Saudi healthcare system [3, 4], but only recently has it achieved prominence as a profession with international recognition of education programmes, professional society formation, and a growing in-country trained workforce. This paper is aimed at charting the emergence and providing an in-depth exploration of the profession via a case study design, thus providing contemporary evidence that can be used in planning and policy.

As a background to this investigation, a precis of the professionalization of OT is now presented. OT emerged as a distinct profession in the early 20^th^ century, with the first professional association of OTs, the National Society for the Promotion of Occupational Therapy (NSPOT), developed in New York, in the United States of America (USA) in 1917 [5]. The first meeting of NSPOT followed years of OT practice in the USA and the United Kingdom helping people with a variety of health conditions primarily related to injury, psychiatric illness, the effects of endemic chronic illness, and congenital conditions. These early OT services were often provided in community clinics, field hospitals, rehabilitation services, repatriation hospitals, and psychiatric asylums.

By 1952, OTs had been organised into an international society known as the World Federation of Occupational Therapists (WFOT), becoming a World Health Organisation (WHO) affiliated organisation in 1959 [6]. The WFOT advances the OT profession by fostering collaboration among member associations, promoting standards for education and training, and advancing OT practice and standards internationally [6]. The number of countries with OT recognised by WFOT has increased from 10 countries in 1952 to 107 countries in 2023; 81 have full WFOT membership, 19 have associate membership, and 7 have regional membership [7]. The Saudi Occupational Therapy Association (SOTA) (https://www.sota.org.sa/) achieved full membership in 2016 [5]. This is a significant historical milestone for Saudi Arabia as the profession demonstrates attainment in practice-, education-, and research-related areas including the publication of a Saudi magazine and newsletter [8].

To date, there has been no comprehensive and systematic investigation into the emergence and development of the OT profession in Saudi Arabia. This case study is aimed at achieving such a goal using the Australian Council of Professions' (ACP) definition of a profession to frame the scope of attributes explored. The ACP defines a profession as a disciplined group adhering to ethical standards; possessing specialised knowledge and skills from high-level research, education, and training; and applying this expertise for others' benefit [9]. In addition, professional characteristics of societal status, professional organisation, extensive training, altruism, job autonomy, ethical code adherence, noncommerciality, societal influence, self-regulation, collegiality, and patient/client focus were considered relevant [10, 11].

The aim of this study is thus to delineate the development of in-country Saudi OT services with regard to the professional association, education, employment/practice areas, codes of conduct, in-country visibility and influence, regulatory frameworks, and aspects of practice to reveal insights into the attributes of professionalism previously identified.

## 2. Method

The study used a case study design to provide an evidence-based description of the OT profession in Saudi Arabia [12]. The case study formed part of a study series conducted within a doctoral research programme at the University of Sydney, which had obtained research ethical approval from Taibah University.

The case study approach allows researchers to explain and describe phenomena or events within a real-life, real-time context. When applied to OT in Saudi Arabia, the case study methodology provided insights into education, service delivery, and gaps in the OT profession both in the past and in the present [12]. Hence, the attributes of professionalism previously described could be sought and identified. A case study methodology involves collecting data from multiple sources, including documents, questionnaires, interviews, and observations [13]. The pivotal strength of the case study methodology is that it uses the in-depth exploration and investigation of a phenomenon, allowing for the inclusion of a wide variety of sources using triangulation to converge the findings. In keeping with the case study methodology, multiple data collection methods were used; these were organised into three phases of procedure, with each focused on various sources of information [13].

Phase 1 involved a structured review and narrative synthesis of publications on OT in Saudi Arabia. Seven databases were searched: Medline, Scopus, the Cumulative Index to Nursing and Allied Health Literature (CINAHL), the Excerpta Medica Database (EMBASE), Allied and Complementary Medicine (AMED), the Joanna Briggs Institute (JBI) Evidence-Based Practice Database, and Web of Science (WoS). The date range for the search was 1 January 1990 to 31 December 2021, and it was updated to 25 November 2023. January 1990 was selected, as this marks the start of the Saudi healthcare system as a modern entity [4]. The first search was conducted in January 2022, and a top-up search using the same strategy was performed in November 2023. The search words used were “occupational therap∗” and “Saudi Arabia”, which combine keywords and medical subject headings (MeSH) [14]. For sources to be considered relevant, they had to be about OT or include information about OT education, practice, or the profession in Saudi Arabia.

Phase 2 entailed an audit of “grey literature” about or referring to OT education, practice, research, or the profession in Saudi Arabia. The documents were retrieved from a variety of official sites and included any document available for public inspection or that could be retrieved with the permission of the author/delegate/organisation. A Google Scholar search was conducted initially, using the terms “occupational therapy” and “Saudi Arabia”. A second Google search was conducted using the terms “occupational therapy” and “Saudi Arabia” in English and then in Arabic. The first 100 results were inspected due to the user algorithm that determines the relevancy of the search results. The time span for the search strategy in Phase 2 was consistent with Phase 1. The third data source was information and documents about the OT profession from different authorities that provide OT services in Saudi Arabia as the Saudi Ministry of Health (MoH), the Ministry of Labour and Social Development, the Saudi Commission for Health Specialties (SCFHS), SOTA, and the National Centre for Archives and Records. A fourth source was identified as the tertiary education institutions that offer OT courses in Saudi Arabia. Finally, the websites of relevant global organisations, such as the WFOT, were investigated for any material relating to OT in Saudi Arabia. The first author was the primary investigator for sources and the sole investigator for searches in Arabic, as it is his native language. Any Arabic sources were translated into English for analysis by the team.

Phase 3 involved consultation with in-country experts in OT to explore and discuss the emerging evidence from the peer-reviewed articles and grey literature identified through phases 1 and 2. In addition, consultation provided opportunities to elicit new information and explore emerging areas. The first author conducted these consultations via personal communication in emails and WhatsApp messaging. These three phases were used to provide an in-depth exploration of the OT profession in Saudi Arabia.

### 2.1. Data Analysis

The findings of Phase 1 through to Phase 3 were summarised and synthesized into a narrative description of the history and contemporary status of OT in Saudi Arabia. Information from all the sources was catalogued and, where appropriate, aggregated to form numerical summaries of the attributes of the sources and the participants. Descriptive numerical data were extracted and collated to provide a summary, and the sources were noted. Content analysis was conducted with data relating to concepts and processes to create a summary by coding the data according to keywords and phrases and then analysing and organising the data into categories through systematic steps including coding textual data, identifying recurring themes and patterns, and organising the findings into meaningful categories [15, 16]. The data were then subjected to descriptive coding and interpretation of the quantitative counts of the codes [15, 16]. The primary goal of this analysis was to provide specific information on the development, professional characteristics, and current status of OT in Saudi Arabia (such as employment trends and education programmes) through textual analysis. This is consistent with the purpose of content analysis [15, 16].

### 2.2. Findings

The results were based on diverse types of sources. In Phase 1, the sources were peer-reviewed papers (mostly empirical research) and conference abstracts in English from various databases from 1990 to 2023. These were relevant to OT in Saudi Arabia and provided information about the profession in the context of that country. In Phase 2, the sources were documents, reports, and information in both Arabic and English that related to research and policy about the OT profession in Saudi Arabia. In Phase 3, the sources consisted of personal communications with OTs in Saudi Arabia. The analysis revealed six topics that summarised and synthesized the evidence about OT in Saudi Arabia: emergence of OT in Saudi Arabia, OT education, certification of OTs, OT professional society, scope of OT practice, and challenges for OT in the 2020s.

#### 2.2.1. Emergence of OT in Saudi Arabia

OT is still considered a new speciality [3, 17–22]. Indeed, there is a dearth of information on the early origins and emergence of OT in Saudi Arabia [18]. OT services have existed in Saudi Arabia since the late 1980s and early 1990s [3, 23–26]. The first OT department in Saudi Arabia was started by the Medical Services of the Ministry of Defence [3], and early services were dependent on foreign OTs from Western countries/cultures [23–28], as at that time, there were no in-country OT educational programmes [24].

#### 2.2.2. OT Education

*(1) The Late 1990s and Early 2000s: Training Institution Diploma Programmes*. During the late 1990s and early 2000s, two OT diploma programmes were offered by two different healthcare institutions (centre for healthcare studies and training and health manpower training institution) (see Table 1), but they were discontinued when the foreign OTs who staffed the programmes left Saudi Arabia in the mid-2000s (A. Aldemyati, personal communication, 18 April 2022; M. Almarashi, personal communication, 21 April 2022). The number of graduates from these programmes could not be reliably ascertained, but it was estimated to be “very few.”

These diploma programmes were delivered in English primarily by international academic and clinical practice scholars. Also, these programmes were granted licenses by the SCFHS [29] (A. Aldemyati, personal communication, 18 April 2022; M. Almarashi, personal communication, 21 April 2022; M. Alotaibi, personal communication, 17 April 2022; S. Al Sindy, personal communication, 17 April 2022).

*(2) Mid-2000s and beyond: University Degree Programmes*. The first wave of bachelor's degree OT programmes was established in Saudi universities in the decade between 2008 and 2017 [3, 22]. The date range rather than definitive dates are provided because published reports and public information about when these programmes were established or when they began teaching OT students vary [3, 18, 22]. For example, a programme may be established, but teaching may not commence until sometime later due to staffing, academic approval, or other reasons.

In the decade between 2010 and 2019, seven OT bachelor's degree programmes were commenced and taught at six government universities: King Saud University [30], King Abdulaziz University [31], Princess Nourah bint Abdulrahman University [32], and all three campuses of King Saud bin Abdulaziz University for Health Science [33]. Batterjee Medical College [34], a private college, also provided a degree in OT. Of these, King Saud University was the first [3, 22, 30], as shown in Table 2.

Since 2019, no new OT programmes have commenced in Saudi Arabia, although there are more in the planning and preparation stages, for example, Northern Border University [3], Qassim University [35], and Taibah University, where development of the academic workforce required to teach the programme is underway (the first author is a Taibah University employee).

All OT bachelor's degree programmes have a duration of five years, including the preparatory and internship years. These seven OT bachelor's degree programmes are recognised by the SCFHS [29]. Two of the OT bachelor's degree programmes use curricula that were developed by international universities that have a record of substantial experience in OT curriculum development [33, 36–38]. To date, the WFOT has officially accredited three of these OT bachelor's degree programmes: those offered by King Saud bin Abdulaziz University, Princess Nourah bint Abdulrahman University, and Batterjee Medical College [39]. To be accredited, courses need to demonstrate the attainment of specific content and attributes [40].

The OT programmes in Saudi Arabia are delivered on a full-time, on-campus basis in the English language. Students who attend government universities do not pay any fees, while private universities expect students to cover the cost of their education, either through self-financing or with the help of a scholarship. No postgraduate OT degree courses are currently available in Saudi Arabia, but the government funds master's and doctoral studies in OT overseas for eligible and interested Saudi graduates. There are no statistics on the number of OTs who have graduated from Saudi bachelor's degree programmes.

#### 2.2.3. Certification of OTs

All health training programmes, as well as practitioner registration and accreditation, are overseen by the SCFHS (https://scfhs.org.sa/en). The SCFHS is responsible for holding licensure examinations for local and foreign nationals to ensure that they can meet the required practice standards and provide high-quality care [29].

The SCFHS classification for OT certification in Saudi Arabia has four categories:

- OT professionals who were awarded a diploma degree in OT from health institutes and colleges are classified as OT technicians. This applies to people who received their OT diploma qualification from abroad
- OTs who have a BSc degree from a College of Applied Medical Science or College of Medical Rehabilitation Science recognised by the SCFHS are classified as OT specialists
- OTs who have an MSc in OT recognised by the SCFHS and have two years of clinical OT experience are classified as OT senior specialists
- OT consultants must have a PhD degree recognised by the SCFHS and three years of clinical OT experience

#### 2.2.4. OT Professional Society

SOTA became a full member of the WFOT after being established for about four years [7]. A professional society is an essential attribute when demonstrating professional status. Professional societies provide a community of colleagues that can offer a focus on advocacy, education, and quality improvement for standards of practice. Current efforts are focused on enhancing practice quality through continuing professional development activities, workshops, and conferences for OT students and therapists. SOTA provides a national point of engagement with the WFOT as a member organisation, and it is able to share information about Saudi practice through the society magazine and newsletter (https://www.sota.org.sa/sota-journal/) [8].

Regarding improving the quality of OT professionals' competencies in clinical practice, the SCFHS designed a postprofessional OT clinical practice programme. It is called the Saudi Diploma of Occupational Therapy (SDOT) but has yet to been activated [41].

#### 2.2.5. Scope of OT Practice

OT services are provided by different governmental health sectors in Saudi Arabia [42, 43] and are mainly provided in secondary and tertiary care to patients with different medical conditions [17, 23–26, 44–50]. According to the latest statistics from the MoH, 91,180 patients received OT services from MoH sectors in 2022 [43]. In comparison, the number of patients who received OT services from other governmental health sectors in 2022 was 59,269. These included medical services provided in the Armed Forces, the National Guard, the King Faisal Hospital Specialist and Research Centres in Riyadh and Jeddah, the Royal Commission Hospitals in Jubail and Yanbu, Aramco Hospital, and the Ministry of Education (teaching hospitals) [43]. Moreover, 16,755 OT sessions were administered at the Care and Rehabilitation Centre for Handicapped Children [43]. OT is a part of home healthcare services [51], and it is also provided in the private sector [52].

OT professionals can make a full assessment, design a care plan, and apply an intervention for patients/clients [18, 53]. However, OT practitioners are only allowed to provide their services to patients following a referral by a physician [18, 19, 24, 48]. The COVID-19 pandemic significantly disrupted the ability to provide OT and rehabilitation services [19].

Moreover, OT services are provided in settings other than hospitals, such as autism centres [52] and the Ministry of Labor and Social Development services [54]. The Ministry of Education's centres provide OT services, such as the Prince Sultan Centre for Special Education Support Services [55]. Also, daycare centres for people with disabilities receive OT services [20]. To date, OT services are not available in primary healthcare settings, community-based services, and schools.

#### 2.2.6. Challenges for OT in the 2020s

The OT profession in Saudi Arabia faces several challenges, which are discussed in the following sections. The five challenges are perceptions and awareness of OT; OT workforce shortages; collaboration with other healthcare professionals; the need for guidelines, standards, and eligibility criteria to practice OT; and the uptake of evidence-based practice.

*(1) Perceptions and Awareness of OT*. There is a scarcity of knowledge about the role of OT in Saudi Arabia [3, 18, 20]. Two studies have assessed OT awareness among healthcare professionals in Saudi Arabia. One study conducted in three hospitals in Makkah City sought to understand how OT is comprehended among healthcare workers, including social workers, physiotherapists, nurses, and physicians [20]. The results indicated a need for more knowledge about the objectives of OT services and associated therapeutic interventions. The results of the study participants who know about OT services are presented in descending order: physicians (52%), physiotherapists (50%), nurses (48%), and social workers (47%). Another study conducted in Al-Ahsa among physicians, allied health professionals (AHPs), and nurses found that healthcare professionals had moderate to minimal knowledge of OT [56]. Consequently, it has been found that most physicians do not refer patients to OT, even if it is available in the hospital [20]. This may be exacerbated by the variability in Arabic and English terms for “occupational therapy” that reflect the nature and role of OT services [57–59].

Sarsak [60] examined perceptions of the OT profession among medical and health science students in Saudi Arabia and found that although 83.2% of students agreed that OT plays a vital role in an interdisciplinary rehabilitation team, they did not know where OTs worked and so needed clarification on the difference between occupational and physical therapy [60].

Another survey asking 4,440 people in Saudi Arabia about their knowledge of OT found that the general public also did not understand the role of OT and its services [61]. In addition, a cross-sectional study compared the knowledge of OT and physiotherapy services between healthcare professionals and the public in Saudi Arabia [62]. Most participants could not distinguish between OT and physiotherapy services, with healthcare professionals having a slightly higher level of understanding than the public [62]. Consequently, there is an urgent need to increase awareness of the OT profession in Saudi Arabia [3, 20, 56, 60–64].

*(2) OT Workforce Shortages*. The WFOT, which conducts a project on human resources every two years, reported a shortage of OTs in Saudi Arabia in clinical settings. The workforce of OT professionals in Saudi Arabia is still limited [3, 18, 19]. The latest report identified that the number of OTs per 10,000 of the Saudi population is 0.1 [52]. The same report showed that 70% of OTs worked in the government sector, while the remaining 30% worked in the private sector [52]. There is no actual national data on the number of OT professionals working in Saudi Arabia [18, 64]. The limited availability of OT education programmes and the shortage of local OTs in Saudi Arabia [18, 19, 64] have led to active recruitment campaigns for international OTs [52]. In addition, the shortage of OT jobs due to variations in the terms used to describe the OT profession has led to the employment of nonspecialists (N. Alshakarah, personal communication, 05 May 2022).

In one study that assessed patient satisfaction with OT services, most patients were satisfied with the OTs and the departmental services they received. However, elderly patients expressed dissatisfaction with appointments and visits as a result of high caseloads and patient volumes in the OT department [44].

*(3) Collaboration with Other Healthcare Professionals*. OT in Saudi Arabia appears to be implemented on a referral basis with limited multidisciplinary teamwork [18, 63]. More collaboration and coordination between physicians and OTs in devising treatment plans would be valuable while the referral-based approach is maintained [18, 24, 48]; thus, there can be clarity and consistency in patient advice. Otherwise, patients will continue to follow the physician's advice instead of the therapist's even if the domain of expertise is within the OT's scope of practice, such as ADL [24]. OT professionals can positively affect the provision of the required OT interventions and treatment planning for hospital patients if they share decisions regarding patients' length of stay [46, 48].

*(4) Guidelines, Standards, and Eligibility to Practice OT*. At present, there is no unified standard or guidelines for OT practice or the scope of OT practice in Saudi Arabia [64]; in other countries, such codes or position statements are issued by professional societies and are used in concert with governmental regulations and codes. Consequently, OTs in Saudi Arabia abide only by the codes of conduct issued by regulatory, licensing, and registration bodies, such as the SCFHS [29]. Since these guidelines can be multi- or interdisciplinary, this can result in confusion regarding how the role of an OT specialist differs from that of other rehabilitation specialities, which may have similar and related aims [61]. Moreover, physiotherapists or special-education specialists who receive 40 hours of OT training can work as OT professionals in day-care centres for people with disabilities, as established in a policy issued by the Ministry of Labour and Social Development [61]. This increases the potential for misunderstanding of the role of OT services [61].

*(5) Uptake of Evidence-Based Practice (EBP)*. Evidence-based practice using research-informed sources is an international standard for effective and high-quality practice in OT [18, 40]. Practitioners need to develop skills and knowledge to implement EBP competencies in their practice, and a shift in values is needed that recognises the importance of research evidence in addition to practice expertise and experience. OTs tend to rely on the opinions of their supervisors or experts rather than research articles and reviews [18]. Indeed, there is little understanding of the extent of evidence-based practice awareness, knowledge, or skill. Therefore, the most significant barriers to implementing EBP include inadequate foundation teaching of EBP in professional preparation or continuing professional development courses, a lack of research knowledge and skills as assumed knowledge for EBP, a lack of interest, a lack of time [18], and communication skill issues [65]. There is a need for encouragement and support for applying EBP within educational and practice settings [18].

## 3. Discussion

The present study is aimed at presenting a timeline and a general picture of the development of the OT profession in academic and clinical settings in Saudi Arabia by analysing and summarising professional status indicators. In addition, issues facing the OT profession in Saudi Arabia were identified. Data were collected from peer-reviewed articles and grey literature as well as the documents, reports, and information available on the websites of relevant organisations relating to the OT profession in Saudi Arabia, and personal communication with in-country experts via emails and WhatsApp messaging to discuss the emerging evidence from peer-reviewed articles and grey literature to elicit new information and discuss opportunities for additional investigation.

Professions are ranked according to the power and prestige they command within the larger society. Thus, true professions are held in high regard and can exert political influence [66–68]. Emerging professions such as OT in some Middle Eastern and African countries, i.e., Jordan [69], Morocco [70], Rwanda [71], and Saudi Arabia [19, 20], are still establishing this regard and influence. Therefore, OT in these countries has slow attributes of professionalization due to a lack of public social acceptance and diminished decision-making autonomy [66–68].

In this case study, OT is still considered a new profession in Saudi Arabia, and OT services are provided mainly in hospitals as a part of rehabilitation services. Despite the growth of OT academic programmes taught in universities, there remain significant areas for further development of the profession [9, 11, 66–68]. Thus, the Saudi OT profession still needs the necessary professional attributes to reflect a true profession for OT, i.e., conferred status and professional autonomy in Saudi Arabia [11, 66–68].

The OT profession's conferred status and services still need to be better enhanced within Saudi society [11, 20, 56, 60, 64, 66–69]. Thus, OT practitioners in Saudi Arabia, like other health professionals, adhere to the codes and regulations of the SCFHS [29]. It is considered part of medical rehabilitation services and is available only in secondary and tertiary care of the healthcare system [72]. OT's professional autonomy is necessary as a separate speciality in healthcare settings or other settings [9, 66–68].

Saudi Arabia's population is expected to increase from 34.8 million in 2020 [73] to 39.5 million by 2030 [74]. Thus, the need for healthcare and OT services will significantly increase [74]. Therefore, the OT profession in Saudi Arabia still demands further development to reflect a true profession in the future as a part of rehabilitation services, as noted by the strategic plan of Saudi Vision 2030 [74]. The following recommendations can be considered based on the analysis of the data in this paper. Firstly, the OT profession in Saudi Arabia needs to be regulated and standardized by establishing a Saudi OT Council. The council will be responsible for national unified OT guidelines and a law of practice that will drive the development of professional education, training, and practice in different areas of OT in the Saudi context [11, 66–68]. It must be also a principal link in the collaboration between all educational sectors and other governmental and private organisations regarding the OT profession and its services in different settings, i.e., health sectors, community-based services, and educational settings. Secondly, enhancing awareness of the OT profession and its services in different settings among the public and health professionals is essential [20, 56, 60, 64, 69]. Thirdly, OT academic programmes should be expanded across the country [3] and postprofessional programmes activated in clinical practice, i.e., the Saudi Diploma in OT [41], following the Saudi Vision 2030, which emphasises aligning educational outcomes with work career expectations and requirements [3, 75]. Fourthly, intensive integrated and developed EBP and related concepts should be included in academic and continuing professional development programmes [76, 77]. Finally, developing and establishing additional models of care to ease access to OT services across Saudi Arabia aligns with the government's vision 2030 blueprint by using broader service design approaches, including a telerehabilitation approach [78, 79].

This case study investigated and explored the history, emergence, and development of the OT profession in Saudi Arabia and the challenges it faces. This study also enhances policymakers' and decision-makers' awareness of the OT profession and its services, which will help resolve the current weaknesses in the OT profession and develop it in the future. However, its limitation was the lengthy process and time spent carrying out this case study because of the various sources used [80].

## 4. Conclusion

Saudi Arabia has experienced gradual growth and development in the OT profession, but it still needs to improve, such as the increased availability and accessibility of required in-person services, which need an increased workforce. Awareness of OT and collaboration between authorities in providing OT services are required to reflect the true nature of this profession and its value to the well-being of the Saudi population, as well as to legislators and healthcare providers. A wide range of OT practices could be expanded, which would make a significant difference in enabling the Saudi Arabian healthcare system and community services to enhance the inclusion and participation of the country's citizens.

## Figures and Tables

**Table 1:** Healthcare institutions with established OT diploma programmes in Saudi Arabia.

Healthcare institutions	Duration	City	Curriculum delivery language	Healthcare institution sector
Centre for healthcare studies and training (Ministry of Defence)	3 years	Riyadh	English	Governmental
Health manpower training institution	2 years and 6 months	Jeddah	English	Private

**Table 2:** Universities with OT bachelor's programmes in Saudi Arabia.

	University name	Commencement date	City	Curriculum delivery language	Duration
1	King Saud University	2010	Riyadh	English	5 years
2	King Saud bin Abdulaziz University for Health Science	2012	Riyadh	English	5 years
3	Princess Nourah bint Abdulrahman University	2013	Riyadh	English	5 years
4	King Saud bin Abdulaziz University for Health Science	2017	Jeddah	English	5 years
5	King Saud bin Abdulaziz University for Health Science	2018	Al-Ahsa	English	5 years
6	King Abdulaziz University	2018	Jeddah	English	5 years
7	Batterjee Medical College	2019	Jeddah	English	5 years

## Data Availability

The published articles, reports, websites, and personal communication data used to support the findings of this study are included within the article.
